# Long-chain polyunsaturated fatty acid lipid and oxylipin alterations in postoperative delirium after cardiac surgery

**DOI:** 10.1016/j.jlr.2025.100959

**Published:** 2025-12-05

**Authors:** Kwame Wiredu, Pruthvi Gowda, James Rhee, Ariel Mueller, Christopher Simon, Occam Kelly Graves, Jason Zhensheng Qu, Matthew Spite, Tina B. McKay, Oluwaseun Akeju

**Affiliations:** 1 Mass General Brigham Department of Anesthesiology, Massachusetts General Hospital, Boston, MA, USA; 2 Department of Cancer Biology, Dana Farber Cancer Institute, Boston, MA, USA; 3 Department of Molecular Metabolism, Harvard T.H. Chan School of Public Health, Boston, MA, USA; 4 Mass General Brigham Department of Anesthesiology, Brigham and Women’s Hospital, Boston, MA, USA

**Keywords:** Alzheimer's disease, cognitive dysfunction, Confusion Assessment Method, dementia, dexmedetomidine, hyperlipidemia, lipidomic, oxidative stress, oxylipin

## Abstract

Lipids play a crucial role in signaling, membrane dynamics, and inflammatory regulation, yet their involvement in postoperative delirium pathogenesis remains unclear. This study examined serum lipidomic alterations in postoperative delirium and assessed the effects of dexmedetomidine treatment on these changes. Lipidomic profiling was conducted at baseline and postoperative day 1 in two independent cohorts of cardiac surgery patients. Mass spectrometry-based shotgun lipidomics and targeted lipid analyses were used to assess lipidomes and oxylipins, respectively. Cardiac surgery was associated with decreased serum lysophospholipids. Postoperative delirium was associated with increased long-chain polyunsaturated fatty acid phospholipids, particularly phosphatidylethanolamines, and elevated oxylipins. Dexmedetomidine, a potential delirium-mitigating medication, reduced long-chain polyunsaturated fatty acid phospholipids. These findings highlight lipid modulation as a potential target for postoperative delirium prevention.

Postoperative delirium is an acute neurobehavioral disturbance affecting arousal, attention, and cognition, occurring in up to 20% of the patients over 60 years old after cardiac surgery (1, 2, 3). It is a distressing complication for patients, families, and caregivers and is associated with long-term cognitive decline, prolonged hospitalization, increased institutionalization, and higher mortality, with estimated annual costs of $32.9 billion (4, 5, 6, 7). While risk factors such as advanced age, sex, frailty, and baseline cognitive function influence susceptibility, the underlying molecular mechanisms remain incompletely understood (5).

Evidence suggests that immune dysregulation plays a central role in postoperative delirium, as elevated levels of inflammatory markers—including C-reactive protein, serum amyloid A, and interleukin-6—have been observed in affected patients (8, 9, 10). Markers of neurodegeneration, such as tau (11, 12, 13), neurofilament light chain (14, 15, 16), and TDP-43 (17), have also been associated with postoperative delirium. Metabolic dysregulation may play a pivotal role in delirium pathogenesis (18, 19), with lipid dysregulation serving as a plausible bridge between inflammation and neurodegeneration.

Lower preoperative levels of unsaturated fatty acids, including n-3 and n-6 polyunsaturated fatty acids and monounsaturated fatty acids, have been associated with postoperative delirium in older patients undergoing hemiarthroplasty for hip-fracture (20). Differential abundance of phosphatidylcholines (PCs) and phosphatidylethanolamines (PEs) and other lipid and metabolites in cerebrospinal fluid have also been reported as risk biomarkers in a similar patient population undergoing knee and hip replacement (21). Omega-3 fatty acids have been reported to improve blood-brain barrier function and reduce the incidence of postoperative delirium in animal models (22, 23) suggesting that lipid profiles may provide insight into their use as risk biomarkers of delirium and as potential therapeutic approaches to promote cognitive resilience to surgical stress.

Lipids are essential for cellular signaling, membrane integrity, and inflammatory regulation, with long-chain polyunsaturated fatty acids (LC-PUFAs) playing a key role in modulating pro-inflammatory pathways (24). Surgical stress and immune activation can trigger significant shifts in lipid composition (25), potentially compromising blood-brain barrier integrity, disrupting metabolic homeostasis, and amplifying neuroinflammatory responses (26). Notably, nighttime dexmedetomidine administration has been associated with a reduced incidence of delirium (27), providing a valuable opportunity to explore the biological plausibility of lipid dysregulation as a contributing mechanism. Specifically, identifying lipidomic changes linked to postoperative delirium and changes effects by dexmedetomidine administration could yield new insights and inform the development of targeted prevention and treatment strategies.

To investigate these relationships, we conducted lipidomic analysis in two cohorts of patients undergoing cardiac surgery with cardiopulmonary bypass (CPB), profiling lipids preoperatively and on postoperative day 1. Our goal was to identify lipid signatures linked to postoperative delirium, validate findings in a second cohort, and evaluate the effect of dexmedetomidine on the lipidome in the setting of postoperative delirium.

## Material and methods

### Study design and ethical approval

This study examined two cohorts of older adults undergoing major cardiac surgery at Massachusetts General Hospital. Study protocols were approved by the Mass General Brigham Institutional Review Board (cohort 1: IRB Protocol #2022P000445; cohort 2: IRB Protocol #2016P000742), and written informed consent was obtained from all participants prior to enrollment. Cases were matched to controls based on age, biological sex, and baseline neurocognitive function, as measured by the telephonic Montreal Cognitive Assessment. The studies were conducted in accordance with the ethical standards outlined in the 1964 Declaration of Helsinki and its subsequent amendments.

Cohort 1 was a nested case-control study derived from a single-site, prospective, observational study investigating postoperative delirium biomarkers in patients undergoing major cardiac surgery with CPB (28). Eligible participants were 60 years or older, scheduled for cardiac surgery with CPB, and had a planned postoperative admission to the cardiac surgical intensive care unit (ICU) for at least 24 h. Exclusion criteria included an ICU stay exceeding two days in the month before surgery, renal or liver failure requiring dialysis or a Child-Pugh score greater than five, severe neurocognitive impairment documented in medical records, SARS-CoV-2 positivity or symptomatic status, blindness, deafness, inability to speak English, or a planned second or emergency surgical procedure during the hospital stay. Delirium assessments were performed using the long-form Confusion Assessment Method at the preoperative visit and twice daily on postoperative days 1 through 3 by trained study personnel.

Cohort 2 was a nested case-control study derived from the randomized, placebo-controlled Minimizing ICU Neurological Dysfunction with Dexmedetomidine-induced Sleep trial, conducted at Massachusetts General Hospital between March 2017 and July 2021 (ClinicalTrials.gov: NCT02856594). The parent study aimed to evaluate the effect of a sleep-inducing dose of dexmedetomidine on the incidence of postoperative delirium in patients undergoing nonemergent cardiac surgery with CPB. Inclusion and exclusion criteria were consistent with those of cohort 1, with the additional exclusion of patients with a documented allergy to dexmedetomidine. Further details of the Minimizing ICU Neurological Dysfunction with Dexmedetomidine-induced Sleep trial protocol and its primary findings have been previously published (12, 27, 29, 30).

### Baseline characteristics

Descriptive statistics on baseline characteristics of study participants are reported as median [Q1, Q3] and count (percentage) for continuous and categorical variables, respectively.

### Patient sample collection

Blood samples were collected using serum separator tubes with clot activator (BD Vacutainer Serum Tubes, Becton, Dickinson and Company, Franklin Lakes, NJ) on preoperative day 0 (0–2 h before surgery) and postoperative day 1. When feasible, blood was drawn from an indwelling line. The serum was isolated by centrifugation at 1,494 × g for 30 min at 22°C, after which the supernatant was aliquoted into cryotubes and stored at −80°C until analysis.

### Lipidomic analysis

#### Shotgun lipidomics

Serum lipid profiling was performed using shotgun lipidomics at Lipotype GmbH (Dresden, Germany) without prior chromatographic separation. One microliter of serum was analyzed using Shotgun Lipidomics platform by Lipotype GmbH, as described previously (31). Internal lipid standards of known concentrations specific to each lipid class were spiked into all samples to enable absolute quantification of measured lipids in picomoles. To ensure data quality, blank samples were included to control for background noise and quality control reference samples were used to assess run-to-run technical variability. Extracted samples were directly infused into a high-resolution hybrid quadrupole-orbitrap mass spectrometer with a nano-flow ESI source and analyzed in positive and negative ion modes. Samples were processed in four separate batches, with quality control samples included in each batch to ensure consistency across runs. Details of sample preparation and experimental procedures have been described previously (31).

### Targeted lipidomics

Targeted lipidomic analysis of 400 μl serum was conducted using liquid chromatography-electrospray ionization tandem mass spectrometry at Lipotype GmbH. Internal standards (15-HETE-d8, 9,10-DiHOME-d4, 13-HODE-d4, 14,15-DHET-d11, LTB4-d4, 20-HETE-d6, 8,9-EET-d11, 9,10-DiHOME-d4, 12,13-EpOME-d4, PGB2-d4, PGE2-d4, PGE2-13,14-dihydro-15-keto-d4, PGF2a-d4) (Cayman Chemical, Ann Arbor) of 500 pg each were spiked into all samples to allow for absolute quantification. Methanol was then introduced, followed by centrifugation and pH adjustment. The resulting supernatant was applied to Bond Elute Certify II columns (Agilent Technologies, Santa Clara) for solid phase extraction. The eluate was evaporated on a heating block at 40°C under a nitrogen stream until a solid residue remained. The residues were then reconstituted in 100 μl of methanol/water. Blank samples were included to control for background noise and quality control reference samples were analyzed to estimate technical variability. Lipid fractions were separated using an Agilent 1,290 high-performance liquid chromatography system coupled to an Agilent 6,495 triple quadrupole mass spectrometer with an ESI source. Chromatographic separation was performed on a Zorbax Eclipse Plus C-18 column (2.1 × 150 mm, 1.8 μm) using a gradient solvent system composed of aqueous acetic acid (0.05%) and acetonitrile, with a flow rate of 0.3 ml/min and an injection volume of 20 μl. Lipid species were detected in negative ion mode using multiple reaction monitoring, with at least two mass transitions recorded per compound to ensure specificity. Lipids with an intensity 5-fold above the blank samples were included in the analysis. The median coefficient of variation of the quality control reference sample was 5.6% across all classes. All samples were analyzed in multiple batches, with quality control samples included in each batch to ensure consistency and reproducibility across runs.

### Data filtering and preprocessing

Noninformative lipid and oxylipin species, including those with values near the detection limit or exhibiting minimal variability across samples, were removed to ensure robust data analysis (32, 33, 34). A relative standard deviation threshold of >25% and an interquartile range threshold of 10% were applied to filter out these species. For parametric statistical analyses, the data were assumed to follow a normal distribution with homogeneous variance and were fitted to a near-Gaussian probability distribution, a standard practice in untargeted metabolomics studies (32, 33, 34). Auto-scaling (unit variance scaling) was applied by mean-centering and dividing each lipid species by its standard deviation, reducing the influence of highly abundant metabolites in multivariate analyses, which is particularly effective for biofluid specimens (32).

### Principal component analysis and data visualization

Principal component analysis (PCA) was performed to evaluate group patterns, with statistical significance assessed using PERMANOVA in MetaboAnalyst 6.0 (34). Euclidean distance based on principal components was used to compute distributions. Heatmaps were generated using Euclidean distance and Ward clustering methods in MetaboAnalyst 6.0 to visualize lipid and oxylipin patterns among preoperative and postoperative patients. The top 150 differentially expressed lipid species (*cohort 1* and *cohort 2*) and the top 50 differentially expressed oxylipin species (*cohort 1*) were considered.

### Meta-analysis of lipidomics data in dexmedetomidine-induced delirium reduction

Meta-analysis was performed using MetaboAnalyst 6.0, with batch effects adjusted via the ComBat method (34). *P*-values from the three datasets were combined using Fisher’s method, with a significance threshold set at 0.05. Differentially expressed lipid species were first identified based on a threshold to generate a list of significant features from each dataset. Each feature’s significance was determined by its occurrence across the datasets, with a minimum of two out of three datasets required for inclusion. An UpSet diagram was generated to visualize the intersection of significant lipid species across datasets.

## Results

### Subject selection and study design

Cases were matched to controls by age, biological sex, and baseline neurocognition. Cohort 1 consisted of 25 patients (16 without delirium, 9 with delirium) and was used to identify lipidomic alterations associated with postoperative delirium. Cohort 2 consisted of 38 patients with delirium (29 no-dexmedetomidine, 9 receiving dexmedetomidine) and 43 patients without delirium (26 no-dexmedetomidine, 17 receiving dexmedetomidine). These data are summarized in Supplemental Tables S1 and S2.

#### Cohort 1

##### Shotgun Lipidomics

**Fig. 1:**
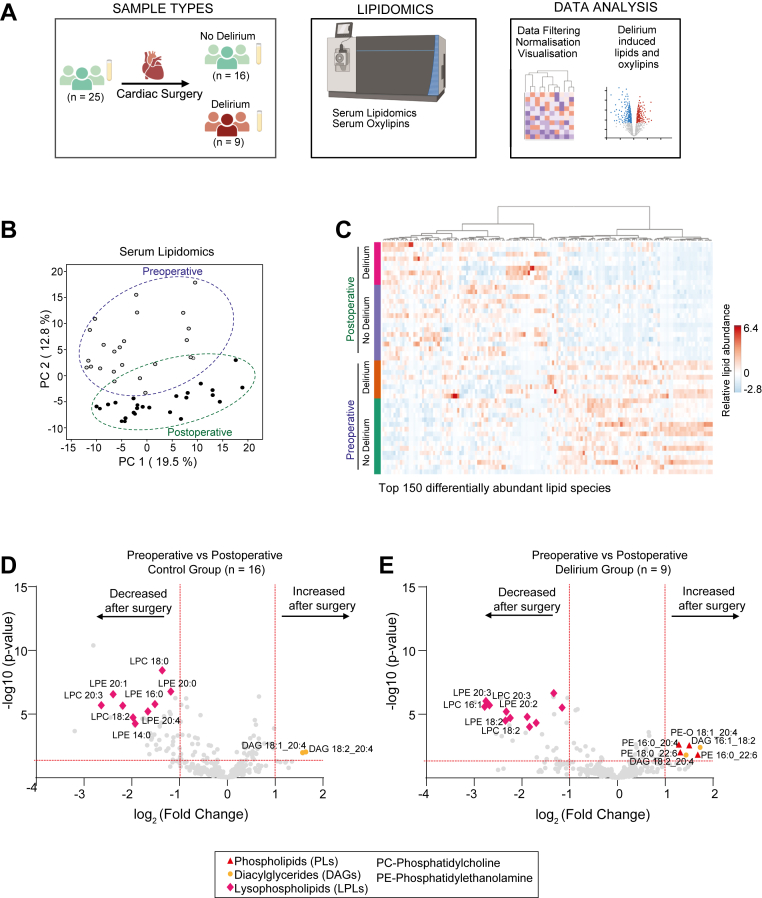
Lipidomic profiling of serum samples from cohort 1. A: Overview of study design. B: Principal component analysis of serum lipidomic profiles in preoperative and postoperative samples. C: Heatmap of the top 150 differentially abundant lipid species stratified by delirium status and timepoint. Red and green dotted boxes highlight clusters of lipid species that increased or decreased, respectively, following surgery. D and E: Volcano plots displaying significantly altered lipid species preoperatively and postoperatively in patients who did not (D) and those who developed delirium (E). Significance thresholds were set at *P* ≤ 0.05 (horizontal red line) and a fold change of ≥2 (vertical red line).

The study design, including sample collection, lipidomic analysis, and data processing, is summarized in Fig. 1A. PCA of serum lipidomic profiles revealed a separation between baseline and postoperative samples. The first two principal components (PC1: 19.5%, PC2: 12.8%) captured a significant proportion of the variance (Fig. 1B). The associated heatmap suggested differential lipid findings in serum collected preoperatively and on postoperative day 1 (Fig. 1C). To identify lipid changes resulting from cardiac surgery, we compared data collected preoperatively and on postoperative day 1 (n = 25) and observed robust decreases in lysophospholipids, particularly lysophosphatidylcholine (LPC) and lysophosphatidylethanolamine (LPE) after surgery. Increases in LC-PUFA-containing diacylglycerols and phospholipids were also observed (Supplemental Fig. S1A). To evaluate delirium-specific lipid changes, we stratified the data into patients who developed delirium (n = 9) and those who did not (n = 16). While no baseline differences in lipid profiles were observed before surgery (Supplemental Fig. S1B), postoperative analysis revealed that patients who developed delirium exhibited a significant increase in LC-PUFA-linked phospholipids, specifically PE, which was not observed in patients without delirium (Fig. 1D, E). No comparable changes were detected in PUFA-linked PCs or phosphatidylinositols (Supplemental Fig. S1C). This included PE species containing LC-PUFA such as arachidonic acid (20:4) and DHA (22:6). In contrast, the decrease in lysophospholipids was present in both groups, suggesting it is a general effect of cardiac surgery rather than specific to postoperative delirium.

##### Targeted Oxylipin Measurements

**Fig. 2:**
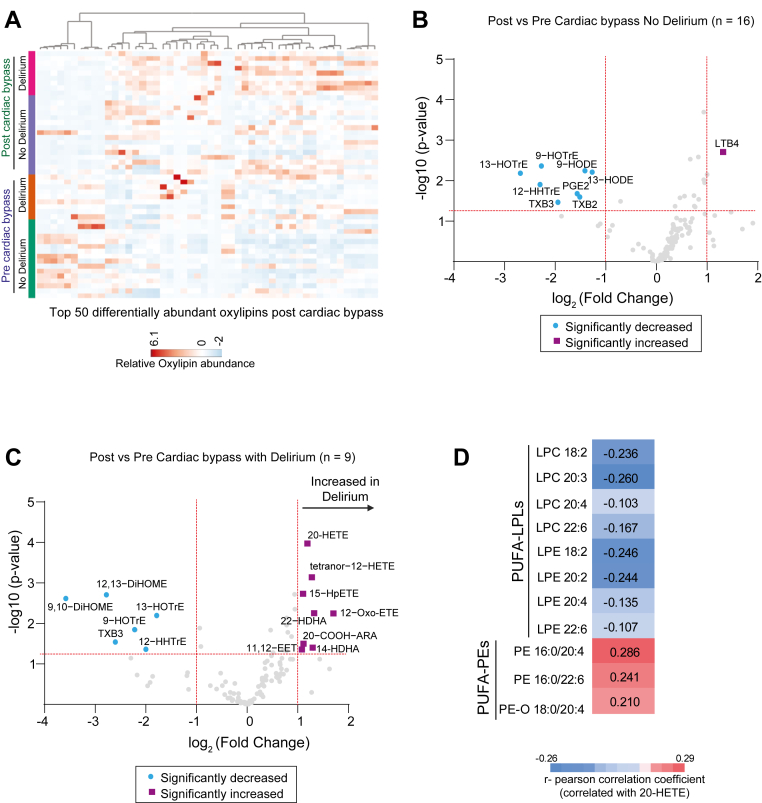
Differential serum oxylipins in delirium and nondelirium groups postcardiac surgery. A: Heatmap of the top 50 differentially abundant oxylipins stratified by delirium status and surgical timepoint. B and C: Volcano plots displaying significantly altered oxylipins preoperatively and postoperatively in patients who did not (B) and those who developed postoperative delirium (C). Significance thresholds were set at *P* ≤ 0.05 (horizontal red line) and a fold change of ≥2 (vertical red line). (D) Pearson correlation coefficients between 20-HETE and select PUFA-LPS and PUFA-PEs.

Given the delirium-associated increase in serum LC-PUFA, we investigated whether oxylipin levels were altered in patients who developed delirium. Oxylipin measurements revealed significant changes on postoperative day 1 after cardiac surgery, with multiple species showing differential regulation in patients who developed delirium (Fig. 2A). At baseline, patients who later developed delirium had slightly higher levels of oxidized short-chain polyunsaturated fatty acids, such as dihydroxy-octadecenoic acid (DiHOME) and prostaglandin products (Supplemental Fig. S2). Compared to patients who did not develop postoperative delirium (Fig. 2B), patients who developed delirium exhibited robust increases in oxidized long-chain polyunsaturated lipids, including HETEs (oxidized arachidonic acid, 20:4) and oxidized hydroxydocosahexaenoic acid (HDHAs, 22:6) (Fig. 2C). For example, increases in cytochrome P450 omega-hydroxylation product 20-HETE and its metabolite 20-COOH-ARA, as well as an omega-hydroxylation product of DHA (i.e., 22-HDHA) were observed. Increased lipoxygenase products including 15-HpETE and 14-HDHA were also observed. These changes were specific to delirium, as similar increases were not observed in patients who did not develop delirium. However, both groups showed a decrease in polyunsaturated fatty acid oxidation products, such as DiHOME and hydroxyoctadecatrienoic acid (oxidized linolenic acid, 18:3), suggesting that these changes are a general effect of cardiac surgery rather than delirium-specific (Fig. 2B, C). Interestingly, oxylipins such as HETEs were negatively correlated with lysophospholipids and positively correlated with LC-PUFA-linked PEs, suggesting a plausible connection between the increase in LC-PUFA-PEs and elevated oxylipins observed in patients with postoperative delirium (Fig. 2D).

#### Cohort 2

**Fig. 3:**
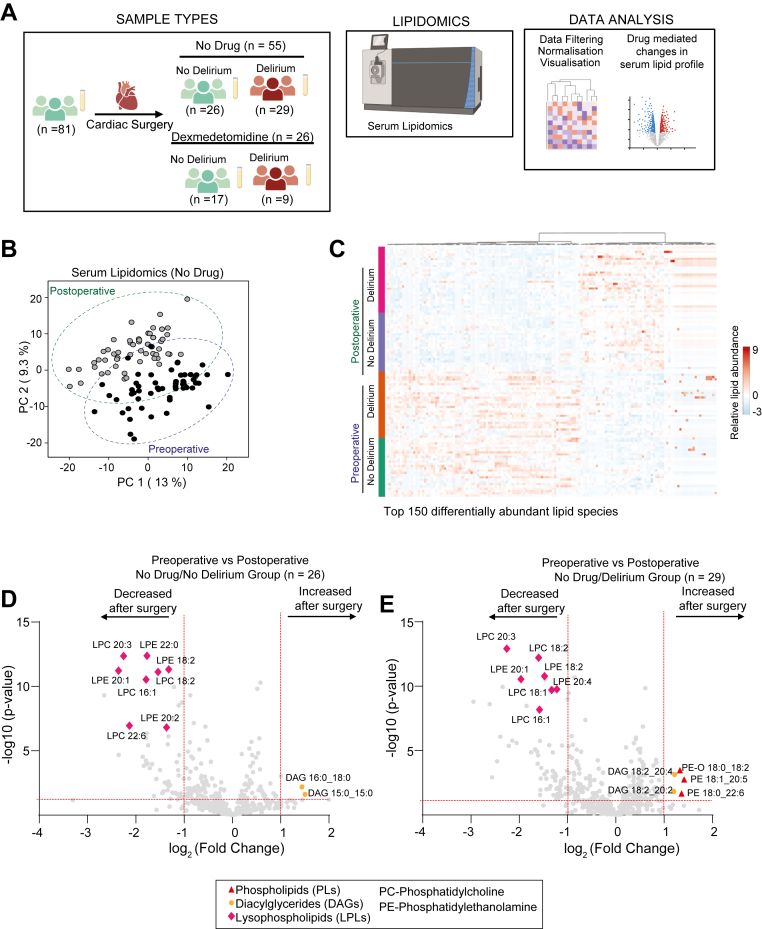
Lipidomic profiling of serum samples from cohort 2. A: Overview of study design. B: Principal component analysis of serum lipidomic profiles in preoperative and postoperative samples. C: Heatmap of the top 150 differentially abundant lipid species. Data analyzed here were from patients that did not receive dexmedetomidine. Red and green dotted boxes highlight clusters of lipid species that increased or decreased, respectively, following surgery. D-E: Volcano plots displaying significantly altered lipid species preoperatively and postoperatively in patients who did not (D) and those who developed delirium (E). Significance thresholds were set at *P* ≤ 0.05 (horizontal red line) and a fold change of ≥2 (vertical red line).

The study design, including sample collection, lipidomic analysis, and data processing, is summarized in Fig. 3A.

### Validation of delirium-induced lipid changes in the second independent cohort

PCA of serum lipidomic profiles in *cohort 2* demonstrated a separation between baseline and postoperative samples. The first two principal components (PC1: 13%, PC2: 9.3%) captured a significant proportion of the variance (Fig. 3B). The associated heatmap illustrated the differential lipid profiles in serum collected preoperatively and on postoperative day 1 (Fig. 3C). Consistent with *cohort 1* (Fig. 1D), cardiac surgery was associated with decreased lysophospholipids, particularly LPC and LPE, alongside increased LC-PUFA-containing diacylglycerols, regardless of postoperative delirium status (Fig. 3D, E, Supplemental Fig. S3A). No baseline differences in lipid profiles were observed prior to surgery (Supplemental Fig. S3B). However, patients who developed delirium exhibited a significant increase in LC-PUFA-linked phospholipids, specifically PE (Fig. 3D and E). Similar to *cohort 1*, the postoperative analysis revealed decreases in lysophospholipids, particularly LPC and LPE, alongside increases in LC-PUFA-containing diacylglycerols, regardless of delirium status.

Thus, increased LC-PUFA-linked phospholipids was observed in both cohorts in patients who developed delirium after cardiac surgery (Fig. 1, Fig. 2, Fig. 3).

### Effect of dexmedetomidine on serum long-chain polyunsaturated lipids

**Fig. 4:**
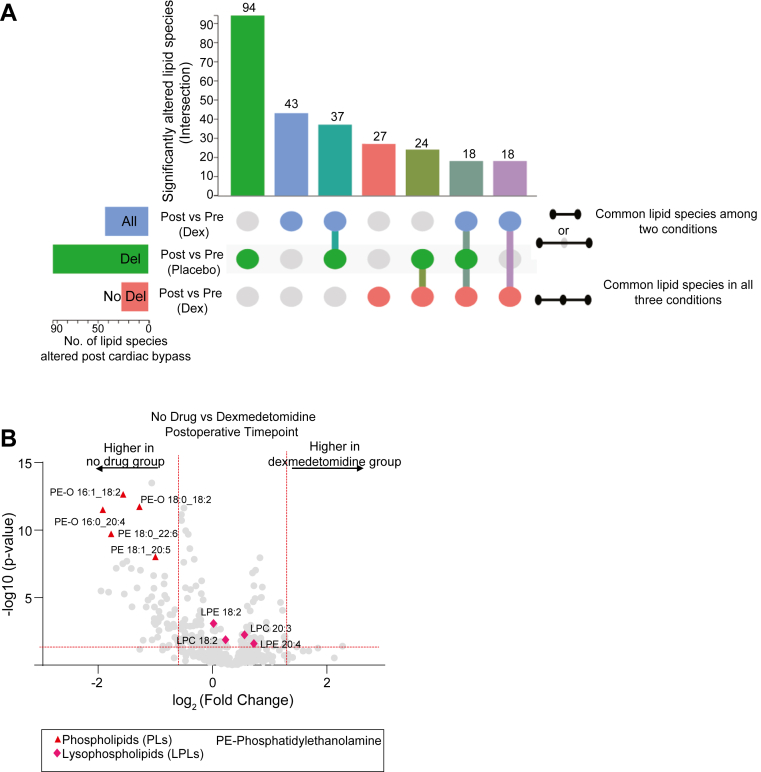
Differential lipidomic responses to dexmedetomidine treatment. A: Upset diagram illustrating the significantly altered serum lipid species across various groups. *P*-values from the three datasets were combined using Fisher’s method, with a significance threshold set at *P* ≤ 0.05. B: Volcano plot of key differentially abundant lipid species between the dexmedetomidine and placebo groups at the postoperative timepoint. Significance thresholds were set at *P* ≤ 0.05 (horizontal red line) and a fold change of ≥2 (vertical red line).

Given the previously demonstrated delirium-mitigating effects of dexmedetomidine in the clinical trial from which cohort 2 was obtained, (27) we next investigated whether dexmedetomidine had any effect on lipid metabolism. A meta-analysis of changes in lipid profiles was conducted across three groups: group 1 comprised patients who developed delirium and did not receive dexmedetomidine (n = 29), group 2 comprised patients who received dexmedetomidine (n = 26), and group 3 comprised patients without delirium that received dexmedetomidine (n = 17). This analysis revealed that the number of serum lipid species significantly altered in delirium in the group was reduced in patients who received dexmedetomidine. Indeed, patients who received dexmedetomidine and did not develop delirium demonstrated the least altered lipid profile (Fig. 4A). Notably, dexmedetomidine was associated with relative reductions in LC-PUFA-linked phospholipids (e.g., PE 18:1; 20:5 and PE 16:0; 20:4) that were elevated in patients with delirium (Fig. 4B).

In summary, cohort 1 demonstrated increases in specific serum LC-PUFA-linked phospholipids and their oxidation products are associated with delirium after cardiac surgery. In the independent cohort *2*, we confirmed that increased serum LC-PUFA-linked phospholipids were associated with delirium after cardiac surgery and further showed that dexmedetomidine, a potential delirium-mitigating medication, attenuated this increase.

## Discussion

Identifying serum biomarkers of postoperative delirium is crucial for advancing our understanding of its pathophysiology, enabling improved risk stratification and the development of targeted therapies (35). In this study, we found that LC-PUFA-linked PEs and their oxidation products were elevated in patients who developed delirium after cardiac surgery. Additionally, baseline alterations in lipid mediators, including decreased 12-HETE and increased 12,13-DiHOME, 9,10-DiHOME, PGB3, and PGJ2, suggest that patients who later develop delirium have a preexisting state of altered lipid metabolism. These baseline differences could reflect underlying metabolic or inflammatory processes that contribute to postoperative delirium. Notably, dexmedetomidine, a potential delirium-mitigating medication, attenuated the increase in LC-PUFA-linked PEs. Collectively, these findings suggest that LC-PUFA-dependent pathways may serve as biomarkers for postoperative delirium and provide insights for therapeutic interventions.

The delirium-associated increase in PE and PE-O species suggests disruptions in cellular membrane dynamics and lipid remodeling in postoperative delirium. Notably, the consistent elevation of PE 18:0/22:6, a DHA-rich phospholipid, across both cohorts highlights DHA’s potential role in delirium pathophysiology. Conversely, the increase in DHA-containing phospholipids in systemic circulation may actually reflect a compensatory response, given DHA’s neuroprotective effects (25, 36). LC-PUFA-enriched phospholipids, including those containing arachidonic acid and DHA, serve as precursors for bioactive oxylipins generated through cyclooxygenase, lipoxygenase, and cytochrome P450 pathways (37, 38). Consistent with this, we observed increased levels of 20-HETE, a pro-inflammatory eicosanoid linked to vascular dysfunction (39), alongside elevated 14-HDHA, a proresolving lipid mediator that may counteract inflammation (40). Although measured peripherally, these oxylipins can influence inflammatory responses within the central nervous system (41). Our prior studies have revealed elevated tau, neurofilament light chain, and TDP-43 on postoperative day 1 in subjects who develop postoperative delirium suggesting links between cognitive changes and markers of neurodamage (17, 28, 42, 43). Lipoproteins in blood are the major carrier and source of LC-PUFA for the brain (44) and their peripheral abundance including the APOE genotype may influence lipid distributions (45). It is important to note that our findings do not preclude the involvement of other mechanisms such as oxidative stress, disrupted membrane remodeling, and altered signaling pathways, which could act in concert with or independently of hypothesized LC-PUFA-mediated effects.

Our analysis revealed significant lipid profile alterations following cardiac surgery in both cohorts, notably a general decrease in LPC and LPE species. This broad shift in metabolism suggests that cardiac surgery-induced stress disrupts cellular membrane remodeling, lipid signaling, and phospholipid homeostasis, potentially driven by increased phospholipase activity and inflammatory lipid turnover. Consistent with prior studies, reduced LPC have been described in septic patients (46, 47), and both LPC and LPE were also reduced in murine sepsis studies (48, 49). Notably, the two patient cohorts in our study both exhibited decreases in LPC 18:2, LPC 20:3, and LPE 20:1, indicating a shared disruption in the pathways involving these specific lysophospholipids. Decreased LPC levels can be caused by a combination of factors: reduced production from PC due to lower lecithin-cholesterol acyltransferase activity, increased conversion back to PC due to higher acyl-CoA:LPC acyltransferase activity, increased breakdown by phospholipase A2, or impaired transport and stability due to low albumin (48). Additionally, both cohorts showed an increase in diacylglycerol species, which may lead to the activation of protein kinase C-mediated signaling pathways (50).

This study has several limitations. First, while we observed significant associations between specific lipid mediators and postoperative delirium, the cross-sectional design precludes definitive conclusions about causality. However, some oxidized LC-PUFAs are known contributors to neurocognitive decline in Alzheimer’s and related disorders (41, 51), suggesting a potential mechanistic link. Second, our study focused on patients undergoing cardiac surgery, which may limit the generalizability of our findings to other surgical populations with differing inflammatory and physiological stress responses. Moreover, serum was studied rather than plasma, which could have skewed the postsurgical profile due to platelet activation. The use of serum may also have influenced oxylipin stability and profiles attributed the coagulation process (52). Third, while we identified significant changes in LC-PUFA-linked phospholipids and oxylipins, the precise functional consequences of these alterations on neuroinflammation, blood-brain barrier integrity, and synaptic function remain to be fully elucidated. Of note, investigating levels of C-reactive protein and other inflammatory proteins in further studies may provide insight into the link between inflammatory factors and these lipid mediators. We also did not detect a statistically significant association between lipid species and biological sex or body mass index after adjusting for multiple comparisons, which will likely require a larger sample size to identify differences. Evaluating other confounders, including genetic factors and ApoE genotype, is warranted. Finally, although we found that dexmedetomidine is associated with a relative reduction in LC-PUFA-linked phospholipids in the systemic circulation, the extent to which this effect shifts the balance between pro-inflammatory eicosanoids and specialized proresolving mediators that have neuroprotective effects (25), requires further investigation. Our findings are expected to promote the development of hypotheses to be tested in further studies regarding the mechanisms involved in dexmedetomidine’s neuroprotective properties.

In conclusion, postoperative delirium after cardiac surgery is strongly associated with increased LC-PUFA levels, with altered phospholipid metabolism and heightened oxylipin production likely playing a central role in its pathophysiology. The attenuation of LC-PUFA-linked phospholipids by dexmedetomidine reinforces the notion of LC-PUFA involvement in postoperative delirium and suggests a lipid-mediated pathway for its protective effects. Furthermore, baseline oxylipin differences indicate a preexisting susceptibility to neuroinflammation and metabolic dysregulation in patients who develop delirium. These findings enhance our understanding of delirium’s metabolic basis and highlight lipid modulation as a potential target for risk stratification and prevention.

## Data Availability

The raw data is available from the Corresponding Author upon reasonable request. The supplemental material includes the comparative analyses between groups.

## Supplemental data

This article contains supplemental data.

## Conflicts of Interest

O. A. is a consultant and holds equity in Reversal Therapeutics. All other authors have no relevant disclosures.

## Supplemental data

All Volcano
