# Supplementary material for: Long-chain polyunsaturated fatty acid lipid and oxylipin alterations in postoperative delirium after cardiac surgery
Source: J Lipid Res. 2025 Dec 5;67(1):100959. doi: 10.1016/j.jlr.2025.100959 (PMC12796732; doi:10.1016/j.jlr.2025.100959)
Supplement: Supplemental Figure S1 [file mmc2.pdf]

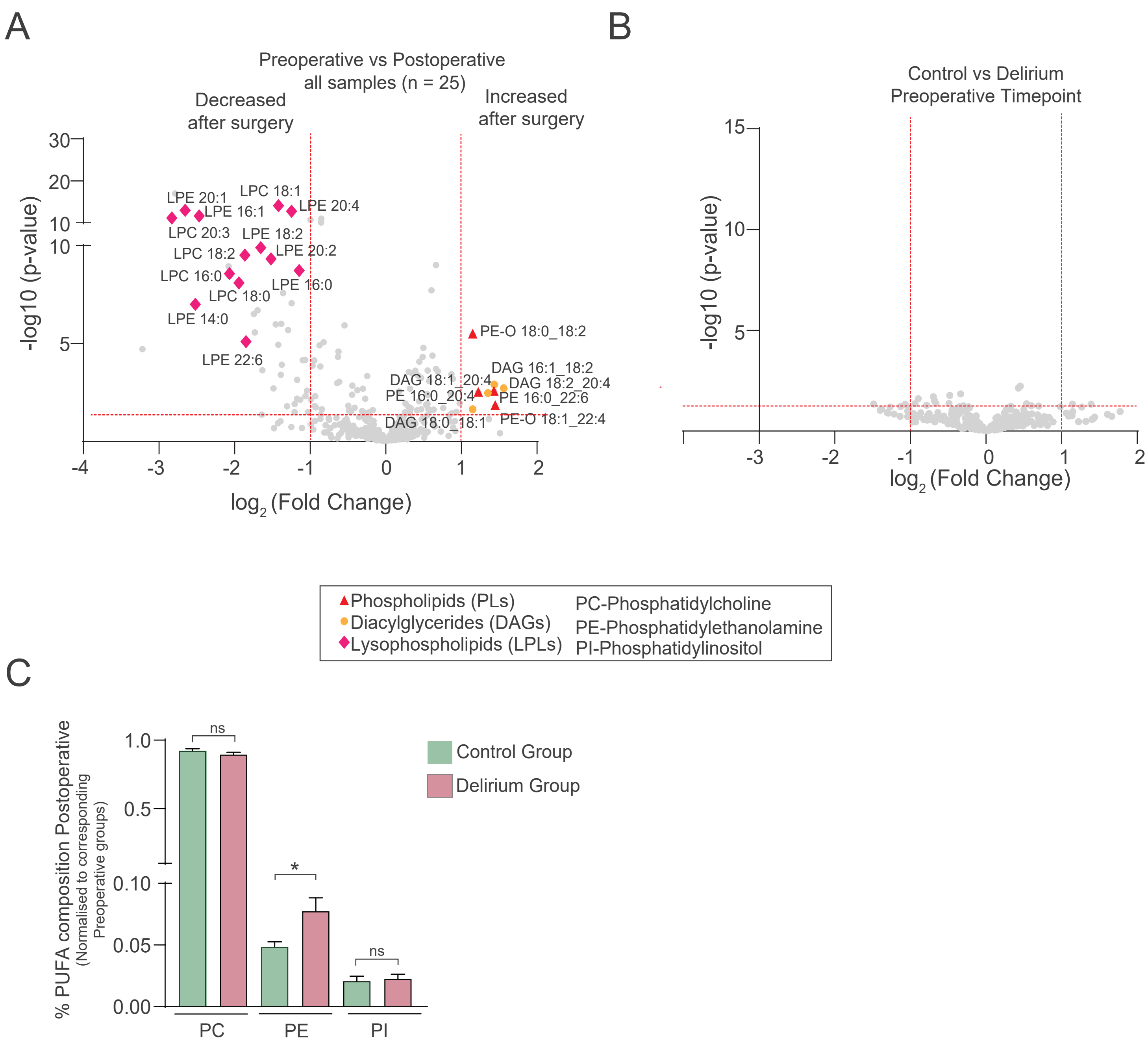

Supplementary Figure 1. Global Serum Lipidomic Alterations Post-Cardiac Surgery from Cohort
(A) Volcano plot illustrating differentially expressed lipid species pre- vs. postoperatively across all study participants. Significance thresholds were set at $p \leq 0.05$ (horizontal red line) and a fold change of ≥2 (vertical red line).
(B) Volcano plot illustrating there were no differences in lipid profiles preoperatively. The same significance and fold change thresholds were applied as in (A).
(C) Bar graph representing the changes in different PUFA linked phospholipids in postoperative control and delirium groups the values are normalised to corresponding preoperative groups.
