# Supplementary material for: Long-chain polyunsaturated fatty acid lipid and oxylipin alterations in postoperative delirium after cardiac surgery
Source: J Lipid Res. 2025 Dec 5;67(1):100959. doi: 10.1016/j.jlr.2025.100959 (PMC12796732; doi:10.1016/j.jlr.2025.100959)
Supplement: Supplemental Figure S2 [file mmc3.pdf]

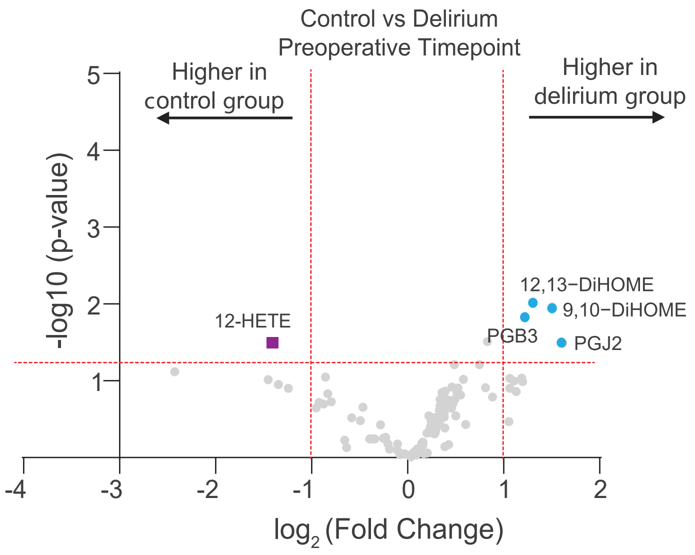

Supplementary Figure 2. Oxylipin profiling of serum samples from Cohort 1. Volcano plot illustrating key differences in oxylipin profiles preoperatively between the delirium and no delirium groups. Significance thresholds were set at $p \leq 0.05$ (horizontal red line) and a fold change of ≥2 (vertical red line).
