# Supplementary material for: Long-chain polyunsaturated fatty acid lipid and oxylipin alterations in postoperative delirium after cardiac surgery
Source: J Lipid Res. 2025 Dec 5;67(1):100959. doi: 10.1016/j.jlr.2025.100959 (PMC12796732; doi:10.1016/j.jlr.2025.100959)
Supplement: Supplemental Figure S3 [file mmc4.pdf]

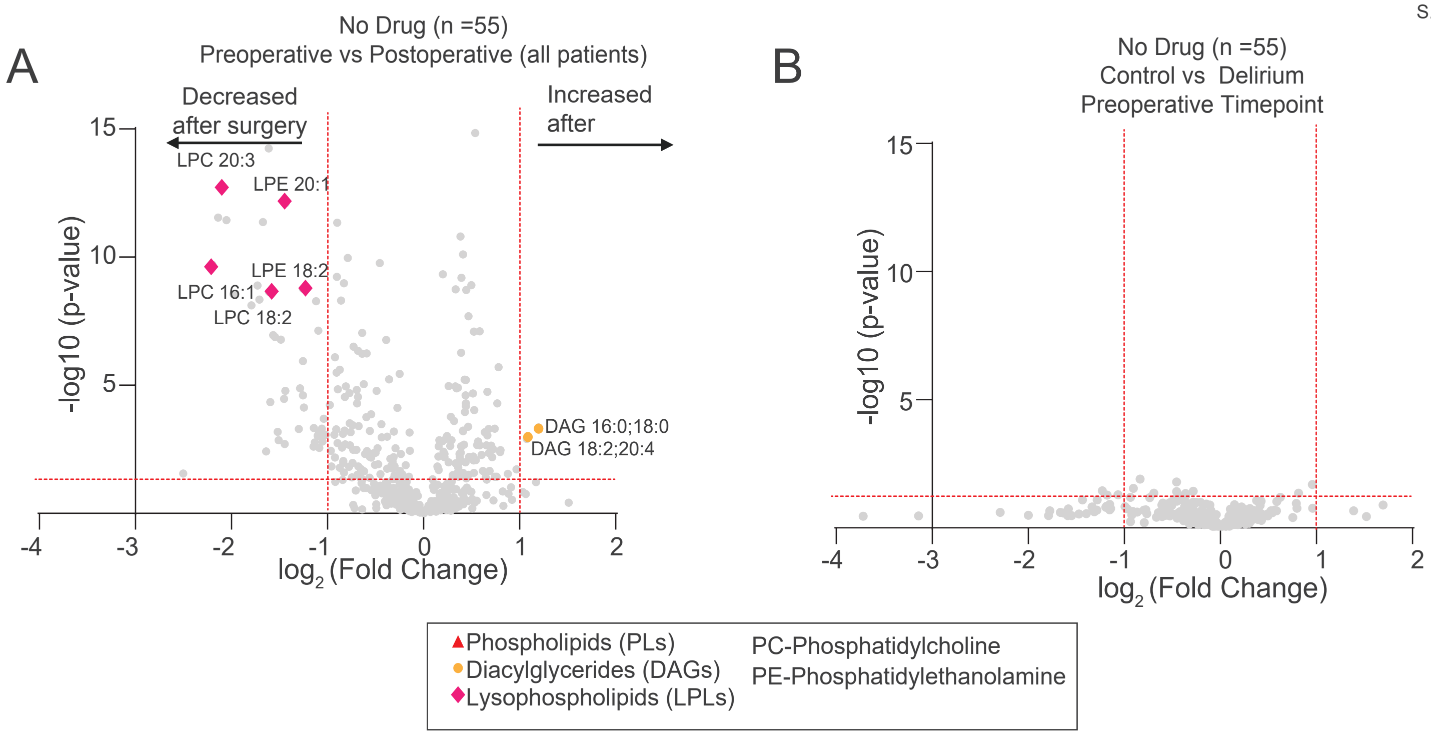

Supplementary Figure 3. Lipidomic profiling of serum samples from Cohort 2.
(A) Volcano plot illustrating significantly altered serum lipid species in post-cardiac surgery. Data analyzed were from patients who did not receive dexmedetomidine. Significance thresholds were set at $p \leq 0.05$ (horizontal red line) and a fold change of ≥2 (vertical red line).
(B) Volcano plot illustrating there were no differences in lipid profiles preoperatively. The same significance and fold change thresholds were applied as in (A).
