# Supplementary material for: Long-chain polyunsaturated fatty acid lipid and oxylipin alterations in postoperative delirium after cardiac surgery
Source: J Lipid Res. 2025 Dec 5;67(1):100959. doi: 10.1016/j.jlr.2025.100959 (PMC12796732; doi:10.1016/j.jlr.2025.100959)
Supplement: Supplemental Table [file mmc5.docx]

| **Supplemental Table 1.** | **Total** | **No Delirium** | **Delirium** |
| --- | --- | --- | --- |
| **Cohort 1** | **n = 25** | **n = 16** | **n = 9** |
| Age (yrs) | 72 [69, 76] | 72 [67, 75] | 72 [71, 76] |
| Sex (male) | 18 (72) | 12 (75) | 6 (67) |
| tMoCA (score) | 19 [17, 21] | 19 [18, 20] | 18 [15, 21] |
| Highest CAM-S (days 1-3) | 3 [3, 8] | 3 [2, 3] | 9 [7, 9] |
| Bypass Duration (mins) | 177 [126, 219] | 177 [122, 206] | 192 [148, 268] |
| **Cohort 2** | **n = 81** | **n = 43** | **n = 38** |
| Treatment (dex) | 26 (32) | 17 (40) | 9 (24) |
| Age (yrs) | 71 [67, 76] | 71 [66, 74] | 72 [68, 77] |
| Sex (male) | 43 (53) | 23 (53) | 20 (53) |
| tMoCA (score) | 20 [17, 20] | 20 [20, 20] | 18 [15, 20] |
| Highest CAM-S (days 1-3) | 5 [3, 7] | 3 [2, 4] | 7 [6, 8] |
| BMI | 28 [25, 32] | 28 [26, 32] | 28 [24, 32] |
| Bypass Duration (mins) | 126 [98, 163] | 121 [95, 146] | 148 [101, 189] |
| Data is presented as median [Q1, Q3] or frequency (%) depending on variable type. *Abbreviations*: tMoCA: Telephone Montreal Cognitive Assessment, CAM-S: Confusion Assessment Method - Severity, BMI: Body Mass Index | | | |

**Supplemental Table 2.**

|  | **Placebo** | | |  | **Dex** | | |
| --- | --- | --- | --- | --- | --- | --- | --- |
|  | **Total** **(n = 55)** | **No Delirium** **(n = 26)** | **Delirium** **(n = 29)** |  | **Total** **(n = 26)** | **No Delirium** **(n = 17)** | **Delirium** **(n = 9)** |
| **Age (yrs)** | 72 [67, 76] | 70 [67, 75] | 73 [69, 77] |  | 69 [65, 74] | 71 [65, 74] | 69 [65, 73] |
| **Sex (male)** | 30 (55) | 14 (54) | 16 (55) |  | 13 (50) | 9 (53) | 4 (44) |
| **tMoCA (score)** | 20 [18, 20] | 20 [20, 20] | 18 [16, 20] |  | 20 [15, 20] | 20 [19, 20] | 15 [14, 19] |
| **Highest CAM-S (days 1-3)** | 5 [3, 7] | 3 [2, 4] | 7 [6, 8] |  | 4 [3, 6] | 3 [2, 4] | 7 [5, 8] |
| **BMI** | 28 [25, 32] | 28 [26, 33] | 27 [24, 32] |  | 28 [26, 32] | 28 [25, 31] | 29 [27, 32] |
| **Bypass Duration (mins)** | 139 [101, 166] | 132 [100, 150] | 148 [101, 183] |  | 110 [93, 145] | 108 [92, 126] | 125 [97, 201] |
| Data is presented as median [Q1, Q3] or frequency (%) depending on variable type. *Abbreviations*: tMoCA: Telephone Montreal Cognitive Assessment, CAM-S: Confusion Assessment Method - Severity, BMI: Body Mass Index | | | | | | | |
